# Anti‐Neuroinflammation Activity of Essential Oils and Fatty Acids

**DOI:** 10.1002/fsn3.71422

**Published:** 2026-01-09

**Authors:** Zelong Jin, Yige Song, Ahmed Attia Ahmed Abdelmoaty, Feng Lin, Jiujun Li, Xianyang Chen

**Affiliations:** ^1^ Department of Pediatrics Shengjing Hospital of China Medical University Shenyang China; ^2^ Bao Feng Key Laboratory of Genetics and Metabolism Beijing China; ^3^ Department of Neurology, Beijing Chaoyang Hospital Capital Medical University Beijing China

**Keywords:** essential oils, fatty acids, neurodegenerative diseases, neuroinflammation

## Abstract

Neuroinflammation plays a pivotal role in the pathogenesis of neurodegenerative disorders, including Alzheimer's disease (AD) and Parkinson's disease (PD). Current pharmacological interventions, such as NSAIDs and corticosteroids, are limited by systemic toxicity and insufficient efficacy in targeting chronic neuroinflammatory processes. Emerging evidence highlights the therapeutic potential of plant‐derived essential oils and fatty acids in modulating neuroinflammatory pathways through multi‐target mechanisms. Essential oils enriched with terpenoids (e.g., carnosic acid, eugenol) attenuate microglial activation and cytokine production by suppressing NF‐κB and MAPK signaling, while polyunsaturated fatty acids (PUFAs), particularly omega‐3, enhance blood–brain barrier integrity and promote the resolution of oxidative stress via Nrf2‐mediated antioxidant defenses. Preclinical studies demonstrate that these compounds reduce Aβ plaque burden, protect dopaminergic neurons, and ameliorate cognitive deficits in animal models of AD and PD. This review provides a comprehensive evaluation of the multifaceted roles of essential oils and fatty acids in combating neuroinflammation and their potential as therapeutic agents. Furthermore, this sets a foundation for further research into translating these natural products into clinical applications in modulating neuroinflammation and related disorders.

## Graphical abstract

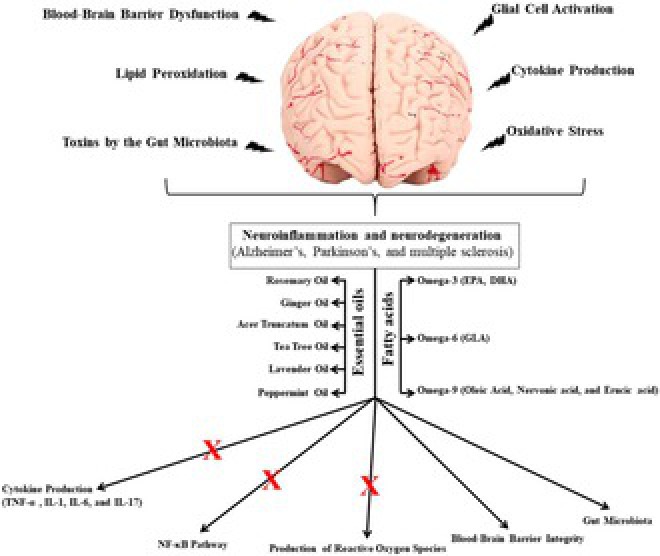

## 1 Introduction

Neuroinflammation is a double‐edged sword in the central nervous system (CNS), serving as a protective response to injury or infection while driving pathological progression in neurodegenerative disorders such as Alzheimer's disease (AD), Parkinson's disease (PD) and Multiple sclerosis (MS) (Kormas and Moutzouri 2022; Morén et al. 2022; Marambaud et al. 2009). Activated microglia and astrocytes release pro‐inflammatory cytokines and reactive oxygen species (ROS), perpetuating neuronal damage and synaptic loss (Kalyanaraman et al. 2024; Pérez and Rius‐Pérez 2022). Despite advances in understanding these mechanisms, current therapies—including NSAIDs and immunosuppressants—exhibit limited efficacy in mitigating chronic neuroinflammation and are often plagued by adverse effects such as gastrointestinal toxicity and compromised immunity (Rivers‐Auty et al. 2020; Aytan et al. 2016; Woodling et al. 2016). This therapeutic gap underscores the urgent need for alternative strategies that target multiple inflammatory pathways with minimal off‐target consequences.

Plant‐derived essential oils and bioactive fatty acids have emerged as promising candidates due to their multiple anti‐inflammatory and antioxidant properties (Lucca et al. 2022; Yan et al. 2023). Essential oils, rich in terpenoids, modulate neuroinflammatory cascades by suppressing NF‐κB and MAPK signaling, thereby reducing microglial activation and cytokine overproduction (Ho et al. 2020; Fanaro et al. 2023). Concurrently, polyunsaturated fatty acids (PUFAs), particularly omega‐3 derivatives (EPA/DHA), stabilize lipid raft dynamics—a critical regulator of membrane receptor clustering and signal transduction—while enhancing blood–brain barrier (BBB) integrity through upregulation of tight junction proteins (Bozzatello et al. 2021). Significantly, lipid raft microdomains, enriched in cholesterol and sphingolipids, serve as platforms for inflammatory receptor assembly, and their disruption has been linked to neurodegenerative pathology (Warda et al. 2025). By targeting these interconnected pathways, natural compounds offer a unique advantage in restoring cellular homeostasis without inducing systemic toxicity.

This review aims to systematically synthesize the current evidence on the anti‐neuroinflammatory mechanisms of essential oils and fatty acids, focusing on their roles in modulating glial activation, oxidative stress, cytokine networks, and BBB integrity. By evaluating preclinical and clinical evidence, we address how these natural products inhibit pro‐inflammatory signaling, enhance antioxidant defenses, and promote neuroprotective responses in models of AD, PD, and MS. Additionally, we highlight translational challenges and future directions, emphasizing the potential of these compounds to bridge the gap between basic research and clinical application in neuroinflammatory diseases.

## 2 Mechanisms of Neuroinflammation

**FIGURE 1:**
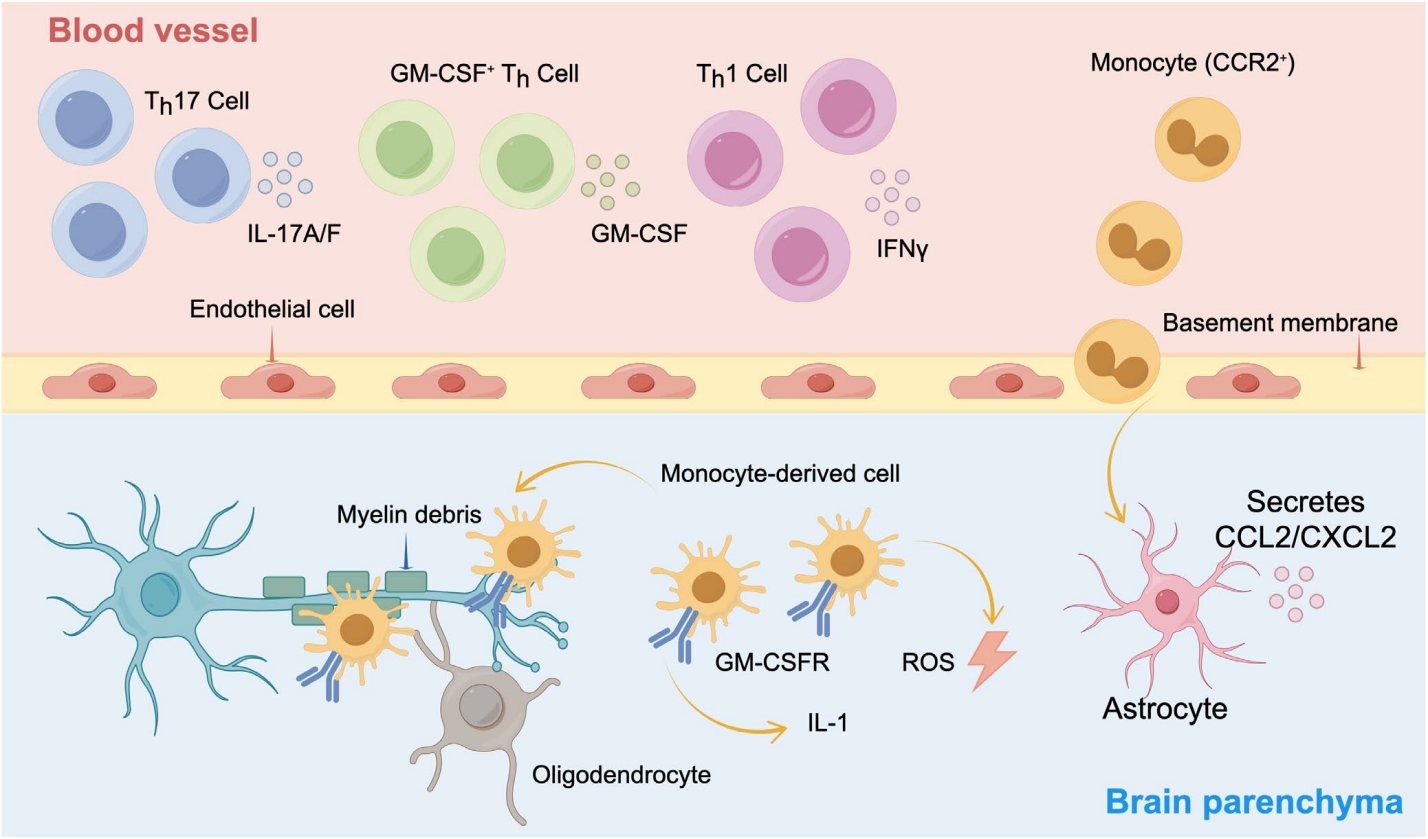
Molecular mechanisms underlying neuroinflammation.

Neuroinflammation in neurodegenerative diseases involves complex interactions between cells and molecules. It starts when T‐helper cells release cytokines, which damage the BBB (Kadry et al. 2020). This damage lets monocytes enter the brain. Monocytes trigger the activation of glial cells, especially astrocytes. Activated astrocytes then release chemokines (Feng et al. 2018; Liu et al. 2023). The monocytes develop into specialized cells that produce reactive oxygen species (ROS), leading to oxidative stress (da Fonseca et al. 2014). Together, glial cell activation, oxidative stress, and BBB damage create self‐sustaining cycles. These cycles ultimately cause neurons to degenerate (Kadry et al. 2020) (Figure 1).

### 2.1 Glial Cell Activation and Inflammatory Signaling

Microglia and astrocytes, the primary immune sentinels of the CNS, exhibit dual roles in neuroinflammation, balancing homeostatic surveillance with pathological damage (Bernaus et al. 2020). In neurodegenerative conditions such as Alzheimer's (amyloid‐β) and Parkinson's (α‐synuclein) diseases, neuronal injury or microbial ligands polarize glial cells toward pro‐inflammatory states (Halle et al. 2008; Lull and Block 2010; Li et al. 2019; Lawrence et al. 2023). M1 microglia activate TLR4/NF‐κB signaling, releasing cytokines (TNF‐α, IL‐1β), chemokines (CCL2), and reactive oxygen species that directly mediate neuronal apoptosis and synaptic loss (Li et al. 2019; Lawrence et al. 2023). Concurrently, A1 astrocytes upregulate complement proteins and matrix metalloproteinases, exacerbating synaptic stripping and extracellular matrix degradation. This dual response disrupts neural circuitry integrity and accelerates neurodegeneration (Liu et al. 2020; Xie et al. 2020).

This glial activation establishes a self‐perpetuating inflammatory loop: microglial ROS production amplifies astrocyte reactivity, while astrocyte‐derived IL‐6 and TNF‐α sustain microglial priming (Bellot‐Saez et al. 2021; Gomolka et al. 2023; Phatnani and Maniatis 2015). In chronic neurodegenerative conditions including AD, PD, and multiple sclerosis, prolonged glial activation shifts the CNS toward a pro‐inflammatory milieu characterized by persistent cytokine elevation and impaired neurotrophic support (Liddelow et al. 2017). In AD, M1 microglia adjacent to amyloid plaques secrete IL‐1β, which enhances Aβ aggregation and tau phosphorylation, while A1 astrocytes in the hippocampus reduce brain‐derived neurotrophic factor (BDNF) expression, contributing to synaptic dysfunction (Halle et al. 2008).

The balance between protective glial states such as M2 microglia and A2 astrocytes, and destructive states like M1 microglia and A1 astrocytes, dictates disease progression (Feng et al. 2018). Therapeutic strategies targeting TLR4/NF‐κB signaling or promoting M2 microglia polarization—such as through interventions with omega‐3 polyunsaturated fatty acids (PUFAs) or essential oils—aim to resolve chronic inflammation and restore neuroprotective glial functions (Rahimifard et al. 2017; Capatina et al. 2021; Postu et al. 2022).

### 2.2 Oxidative Stress and Lipid Peroxidation

Oxidative stress arises from an imbalance between ROS production and antioxidant defenses, with glial cell activation and mitochondrial dysfunction serving as primary drivers (Barnham et al. 2004; Bhatia and Sharma 2021). Mitochondrial electron transport chain dysfunction in neurons and NADPH oxidase activation in microglia and astrocytes generate superoxide anions (O_2_^−^) and hydroxyl radicals (•OH), which exceed the capacity of endogenous antioxidants like glutathione (GSH) and superoxide dismutase (SOD) (Singh et al. 2019). This oxidative burden preferentially targets PUFAs in neuronal membranes, initiating lipid peroxidation—a process that produces cytotoxic aldehydes such as 4‐hydroxy‐2‐nonenal (4‐HNE) and malondialdehyde (MDA) (Singh et al. 2019). These reactive species covalently modify proteins, promoting their misfolding and aggregation, while also damaging DNA and disrupting mitochondrial integrity (Singh et al. 2019).

Iron accumulation in the CNS further exacerbates oxidative stress through Fenton reactions, generating OH radicals and driving ferroptosis—a lipid peroxidation‐dependent form of regulated cell death (Ward et al. 2014). Ferroptosis selectively affects vulnerable neuronal populations in diseases like PD and AD, with studies linking iron‐induced lipid peroxidation to dopaminergic neuron loss in the substantia nigra and hippocampal synaptic dysfunction (Li et al. 2022, 2023; Zhu et al. 2022).

The crosstalk between neuroinflammation and oxidative stress creates a bidirectional pathogenic loop (Wang et al. 2023). Pro‐inflammatory cytokines upregulate NADPH oxidase and mitochondrial ROS production, while ROS in turn enhance glial cell activation via NF‐κB and MAPK signaling (Wang et al. 2022). This vicious cycle is exemplified in AD, where Aβ oligomers induce microglial ROS release, promoting tau hyperphosphorylation and synaptic loss, while astrocyte‐derived IL‐6 exacerbates mitochondrial dysfunction in neurons (Wang et al. 2023).

Natural compounds targeting oxidative stress, such as resveratrol and curcumin, show promise by scavenging ROS, enhancing GSH synthesis, and inhibiting lipid peroxidation (Ashok et al. 2022; Grabarczyk et al. 2024). Curcumin reduces 4‐HNE levels in APP/PS1 mouse brains, correlating with improved cognitive performance, while resveratrol activates Nrf2‐ARE signaling to upregulate antioxidant enzymes in microglia (Su et al. 2022; Cai et al. 2023; Kato et al. 2023). However, translating these findings to clinical settings requires addressing challenges in bioavailability and CNS penetration.

### 2.3 Blood–Brain Barrier Disruption

Neuroinflammatory cascades critically compromise BBB integrity through synergistic mechanisms involving endothelial dysfunction, junctional protein degradation, and lipid raft disorganization (Hawkins and Davis 2005; Schaeffer and Iadecola 2021; Iadecola 2017). The BBB, composed of tightly connected brain microvascular endothelial cells (BMECs), pericytes, and astrocytic end‐feet, dynamically regulates CNS homeostasis via selective permeability (Kadry et al. 2020; Takata et al. 2021). Under neuroinflammatory conditions, activated microglia‐derived TNF‐α and IL‐1β induce RhoA/ROCK‐mediated cytoskeletal contraction in BMECs, reducing transendothelial electrical resistance (TEER) and increasing paracellular flux (Yang et al. 2019). This is mechanistically linked to the downregulation of tight junction proteins (claudin‐5, occludin, ZO‐1) via NF‐κB‐dependent suppression of junctional adhesion molecule‐A (JAM‐A) transcription. Concurrently, reactive astrocytes release matrix metalloproteinases (MMP‐2/9) that proteolytically degrade basal lamina components, further destabilizing BBB architecture (Yang et al. 2019).

Enhanced endothelial permeability facilitates the infiltration of peripheral immune cells and circulating damage‐associated molecular patterns (DAMPs), which amplify neuroinflammation through feedforward cytokine loops. Infiltrating monocytes differentiate into pro‐inflammatory macrophages, secreting IL‐6 and CCL2 that reactivate resident microglia—a phenomenon observed in MS lesions and AD models (Liebner et al. 2018; Xiao et al. 2020; Hussain et al. 2021).

Lipid raft microdomains in BMECs serve as critical regulators of BBB integrity, orchestrating caveolin‐1‐mediated transcytosis and solute carrier (SLC) trafficking (Grassi et al. 2020). Neuroinflammatory insults induce cholesterol efflux via ABCA1 upregulation and sphingomyelinase activation, disrupting raft clustering and impairing P‐glycoprotein (P‐gp) efflux function (Karasinska et al. 2013; Cashikar et al. 2023). This lipid dyshomeostasis reduces insulin receptor substrate‐1 (IRS‐1) localization to rafts, diminishing insulin‐mediated glucose uptake and exacerbating endothelial metabolic stress (Karasinska et al. 2013; Cashikar et al. 2023). Omega‐3 fatty acids (DHA/EPA) restore raft cholesterol content and caveolin‐1 phosphorylation, rescuing Aβ clearance via LRP1 transcytosis in AD models (Lim et al. 2005).

### 2.4 Cytokine‐Driven Neurodegeneration

Sustained production of IL‐6, IL‐17, and IFN‐γ activates JAK–STAT and MAPK pathways in neurons, inducing apoptosis via caspase‐3 cleavage and Bcl‐2 suppression. Simultaneously, TNF‐α potentiates glutamate excitotoxicity by inhibiting astrocytic glutamate uptake, leading to Ca^2+^ overload and neuronal hyperexcitability. These cascades converge to accelerate synaptic loss and gray matter atrophy, as observed in advanced neurodegenerative stages (Rahimifard et al. 2017; Guo et al. 2022; Blevins et al. 2022).

Pro‐inflammatory cytokines orchestrate a dual role in neuroinflammation, transitioning from physiological regulators to pathological drivers when their production becomes sustained or spatially dysregulated. TNF‐α and IL‐1β, initially critical for synaptic pruning and pathogen clearance, undergo context‐dependent functional shifts that exacerbate neurodegenerative cascades (Block et al. 2007; Takeuchi et al. 2006).

TNF‐α signals through two structurally distinct receptors: TNF‐R1 and TNF‐R2. Under neuroinflammatory conditions, lipid raft clustering facilitates TNF‐R1 oligomerization, activating caspase‐8 via the TRADD/FADD complex and inducing neuronal apoptosis. Concurrently, TNF‐α inhibits astrocytic glutamate transporter EAAT2 expression, leading to extracellular glutamate accumulation and NMDA receptor‐mediated Ca^2+^ influx—a mechanism implicated in excitotoxic dendritic spine loss (Guo et al. 2022; Block et al. 2007).

IL‐1β exerts its effects via IL‐1R1/MyD88 signaling, which recruits IRAK4 to phosphorylate IKKβ, driving NF‐κB nuclear translocation and subsequent COX‐2/PGE2 synthesis. In Alzheimer's pathology, IL‐1β enhances β‐secretase (BACE1) activity by modulating γ‐secretase complex assembly within lipid rafts. IL‐1β‐induced MMP‐9 secretion disrupts perineuronal nets, accelerating tau hyperphosphorylation and neurofibrillary tangle formation (Guo et al. 2022; Block et al. 2007).

IL‐6 activates JAK2/STAT3 signaling in neurons, upregulating pro‐apoptotic BIM while suppressing anti‐apoptotic Bcl‐2 (Liu et al. 2022). This imbalance primes mitochondria for cytochrome C release, activating caspase‐3 and inducing DNA fragmentation. IL‐17 synergizes with TNF‐α to enhance ROCK2‐mediated phosphorylation of NF‐L, destabilizing axonal neurofilaments and impairing axonal transport—a hallmark of progressive MS and PD. IFN‐γ exacerbates ER stress through PERK/eIF2α pathway activation, increasing ATF4‐driven CHOP expression that promotes caspase‐12‐mediated apoptosis (Block et al. 2007; Mosley et al. 2006; Gaschler and Stockwell 2017).

## 3 Essential Oils, Fatty Acids and Their Anti‐Neuroinflammatory Mechanisms

### 3.1 Essential Oils

#### 3.1.1 Rosemary Oil

Derived from *Rosmarinus officinalis* , rosemary oil exhibits multifaceted neuroprotective effects mediated by its bioactive constituents, including carnosic acid, rosmarinic acid, and terpenoids (Horváth et al. 2021) (Table 1). Carnosic acid, a diterpenoid, demonstrates potent antioxidant activity by upregulating transcriptional pathways such as Nrf2‐ARE, enhancing the expression of antioxidant enzymes to scavenge ROS and mitigate lipid peroxidation in neuronal cells (Yi‐Bin et al. 2022). Carnosic acid inhibits neuroinflammation, reduces Aβ deposition, and improves cognitive function by targeting the CEBPβ‐NFκB signaling pathway (Yi‐Bin et al. 2022).

**TABLE 1:** Summary of essential oil mechanisms in anti‐neuroinflammation.

Essential oils	Component	Chemical structure	Possible mechanisms of anti‐neuroinflammation	References
Rosemary Oil	Carnosic acid	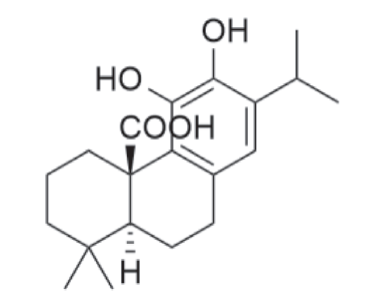	Antioxidant stress, inhibition of neuroinflammation, regulation of Nrf2‐ARE pathway, inhibition of CEBPβ‐NFκB signaling, reduction of Aβ deposition	(Yi‐Bin et al. 2022)
	Rosmarinic acid	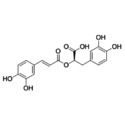	Inhibition of NF‐κB activation, suppression of pro‐inflammatory cytokines, antioxidant stress (enhancement of GSH biosynthesis), anti‐Aβ excitotoxicity	(Yu et al. 2022)
Ginger Oil	Gingerol	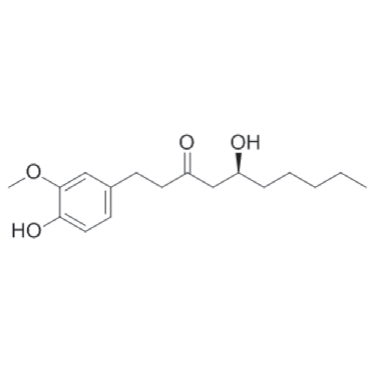	Activation of Nrf2‐Keap1 signaling, inhibition of NLRP3 inflammasome, suppression of NF‐κB activation, reduction of pro‐inflammatory mediators (IL‐1β, NO, iNOS)	(Ball et al. 2020; Sapkota et al. 2019)
	Shogaol	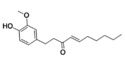	Inhibition of HDAC, induction of HSP70, reduction of pro‐inflammatory cytokines, HDAC‐dependent neuroinflammation attenuation	(Jimbo et al. 2009)
Lavender Oil	Linalool	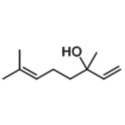	Inhibition of NF‐κB pathway, reduction of pro‐inflammatory cytokines, antioxidant stress, anti‐Aβ accumulation, modulation of cytokine levels	(Szymczak et al. 2024; Stojanović et al. 2024; Aboutaleb et al. 2019; Sabogal‐Guáqueta et al. 2016)
Clove Oil	Eugenol	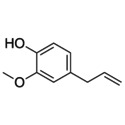	Inhibition of pro‐inflammatory mediators (lipoxygenase, IL‐1β, COX, iNOS), antioxidant stress (enhancement of GSH), anti‐apoptosis, modulation of microglial activity	(Yuan et al. 2021; Del Prado‐Audelo et al. 2021; Holmes et al. 2002)
Acer Truncatum Oil	*Linoleic acid*	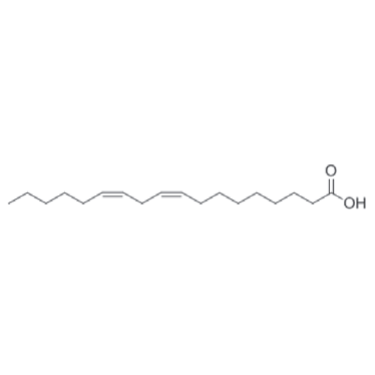	Anti‐inflammatory, antioxidant, neural tissue maintenance, promoting nerve regeneration, modulating neural transmission.	(Fu et al. 2013; Burns et al. 2011; Kumar et al. 2016)
	*Oleic acid*	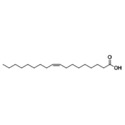		
	*Nervonic acid*	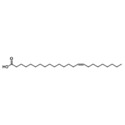		
Tea Tree Oil	Terpinen‐4‐ol	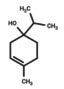	Regulating immune responses, reducing oxidative stress, inhibiting pro‐inflammatory cytokines.	(Ahmad et al. 2012; Qiao et al. 2018)
Peppermint Oil	Menthol	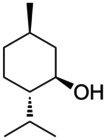	GABAA receptor modulation, AChE inhibition, neurotransmitter regulation, cognitive enhancement	(Song et al. 2022; Chen, Song, Song, Cao, et al. 2023)

Rosmarinic acid, a phenolic acid, complements carnosic acid by targeting both oxidative stress and inflammatory signaling. It suppresses NF‐κB pathway activation by inhibiting IκBα phosphorylation, leading to reduced expression of pro‐inflammatory cytokines (TNF‐α, IL‐1β) and iNOS in LPS‐stimulated microglia. Additionally, rosmarinic acid enhances GSH biosynthesis, restoring redox balance and protecting neurons from Aβ oligomer‐induced excitotoxicity (Yu et al. 2022).

Beyond its antioxidant and anti‐inflammatory roles, rosemary oil modulates neurotransmitter systems and neurotrophic factors critical for cognitive function. It potentiates dopaminergic, serotonergic, and cholinergic signaling in the brain—effects validated in AD rodent models via increased tyrosine hydroxylase (TH) activity and acetylcholinesterase (AChE) inhibition, thereby elevating acetylcholine levels to improve cognition. Concurrently, in vitro and in vivo studies demonstrate that rosemary oil downregulates genes such as β‐amyloid, caspase‐3, and glial fibrillary acidic protein (GFAP), suppressing neuronal apoptosis and glial overactivation (Rezk et al. 2023; Vasdev et al. 2022). These robust preclinical findings provided the rationale for clinical translation. A trial in Alzheimer's patients reported improved cognitive orientation following aromatherapy with rosemary and lemon essential oils (Jimbo et al. 2009). Nevertheless, these findings stand in contrast to a systematic review of 13 RCTs, which found no convincing evidence for the efficacy of aromatherapy on cognition or behavior in dementia (Ball et al. 2020). This discrepancy underscores the necessity for larger and more rigorously controlled clinical trials to definitively assess the therapeutic potential of rosemary oil.

#### 3.1.2 Ginger Oil

Derived from *Zingiber officinale* , ginger oil contains bioactive gingerol and shogaol, which exhibit potent neuroprotective effects via multi‐target mechanisms (Sapkota et al. 2019) (Table 1).

6‐Shogaol protects dopaminergic neurons in PD and MS models by inhibiting histone deacetylase (HDAC) to reduce pro‐inflammatory cytokine expression and induce heat shock protein 70 (HSP70), critical for protein homeostasis (Sapkota et al. 2019). In MPTP‐treated mice, it mitigates nigral neuron loss and improves motor function, linked to reduced α‐synuclein aggregation. Gingerol activates Nrf2‐Keap1 signaling, enhancing antioxidant enzyme expression and GSH levels, while suppressing NLRP3 inflammasome‐mediated IL‐1β release in Aβ‐exposed neurons (Huh et al. 2020).

Ginger extract improves cognitive function in memory‐deficit models, correlating with enhanced synaptic markers in the hippocampus (Mao et al. 2019). 10‐Gingerol, a major homolog, inhibits microglial NF‐κB activation, reducing NO and iNOS production in LPS‐stimulated cells. In MS‐like models, 6‐shogaol attenuates neuroinflammation and cognitive decline via HDAC‐dependent pathways (Mao et al. 2019).

While gingerol's oral bioavailability is moderate, metabolites like 6‐paradol exhibit improved BBB permeability, suggesting gut‐brain axis involvement (Bischoff‐Kont and Fürst 2021). Ongoing trials assess its role in reducing inflammatory biomarkers and supporting neurotrophic signaling in mild cognitive impairment (Szymczak et al. 2024; Stojanović et al. 2024).

Ginger oil's dual targeting of inflammation, oxidative stress, and synaptic integrity positions it as a promising adjunct for neurodegenerative diseases, with future research focusing on delivery optimization and standardized formulations.

#### 3.1.3 Lavender Oil

Lavender oil, extracted from the flowers of *Lavandula angustifolia* , has garnered significant attention in the field of aromatherapy and pharmacology due to its diverse therapeutic properties, particularly those attributed to its active compound, linalool. Studies on the anti‐neuroinflammatory activity of linalool reveal its multifaceted role in mitigating neuroinflammation, particularly in the context of neurodegenerative diseases (Aboutaleb et al. 2019) (Table 1).

The potential of linalool to enhance social interaction behaviors in mice was demonstrated, indicating its influence on neuroinflammatory responses and behavioral outcomes. This foundational research underscored the importance of understanding how linalool interacts with the CNS and its capacity to modulate cytokine levels, which are pivotal in neuroinflammatory processes. Also, authors reported significant improvements in learning and memory in elderly mice treated with linalool, alongside a reduction in pro‐inflammatory markers and Aβ accumulation. This evidence reinforces the notion that linalool can mitigate oxidative stress and inflammation, contributing to its therapeutic potential in the context of age‐related neurodegenerative diseases such as AD and PD (Sabogal‐Guáqueta et al. 2016).

Further investigation demonstrated that linalool significantly reduces inflammation associated with Aβ42‐induced neurodegeneration. The findings established link between neuroinflammation and the progression of AD. Additionally, the authors provided evidence that linalool decreases ROS levels, which are known to contribute to neuronal damage and death in AD (Yuan et al. 2021). Additionally, the inhibition of the NF‐κB pathway as a critical mechanism through which linalool exerts its anti‐inflammatory effects was explored (Del Prado‐Audelo et al. 2021). By inhibiting this pathway, linalool may reduce the expression of pro‐inflammatory cytokines, thereby contributing to its neuroprotective profile.

Despite a compelling mechanistic rationale, the clinical translation of essential oils for dementia has produced inconsistent results. Reports on lavender oil are particularly contradictory. One controlled trial by Holmes et al. demonstrated a significant reduction in agitation in patients with severe dementia (Holmes et al. 2002). In contrast, other studies have found no significant benefits for behavioral symptoms (O'Connor et al. 2013). Illustrating this discrepancy, a randomized controlled trial (*n* = 67) investigating lavender oil combined with massage for behavioral disturbances found no statistical difference between the intervention and placebo groups (Fu et al. 2013). The evidence for other essential oils is similarly unconvincing. A separate double‐blind trial comparing a combination of lemon balm essential oil and donepezil against placebo observed improvements across all groups, with no added benefit attributable to the essential oil itself (Burns et al. 2011). Collectively, these clinical studies indicate that the effectiveness of aromatherapy for dementia‐related symptoms remains unproven, a key finding not adequately reflected in this review. The divergent outcomes likely stem from methodological variations, including differences in patient cohorts, outcome measures, and administration protocols.

#### 3.1.4 Clove Oil

Clove oil, derived from the buds of the *Syzygium aromaticum* plant, is characterized by its high eugenol content, which is responsible for its notable therapeutic properties, including anti‐inflammatory, antioxidant, and analgesic effects. Clove oil has emerged as a potential therapeutic agent in managing neuroinflammation and oxidative stress, particularly in the context of neurodegenerative diseases such as AD, PD, and MS (Kumar et al. 2016) (Table 1).

The exploration of anti‐neuroinflammatory activity through the modulation of cytokines has gained significant attention in recent years, particularly concerning the therapeutic potential of natural compounds such as clove. A previous study investigated the ability of eugenol to inhibit pro‐inflammatory mediators such as lipoxygenase, interleukin 1β, cyclo‐oxygenase, and nitric oxide synthase. This inhibition is significant because inflammation is a well‐known contributor to neurodegenerative processes. By reducing these inflammatory markers, clove oil can potentially protect neurons from the detrimental effects of inflammation, thereby promoting neuronal survival in adverse conditions (Soares et al. 2021).

Another study demonstrated a comprehensive analysis of the neuroprotective properties of eugenol, a primary component of clove oil. This study highlighted eugenol's ability to protect neurons from apoptosis, which is a critical factor in the progression of neurodegenerative conditions such as AD. The article further elaborated on the mechanisms through which eugenol exerts its protective effects, notably its antioxidant and anti‐apoptotic properties. These properties are essential in combating oxidative stress, a key contributor to neuronal damage and degeneration. The findings from this research indicated that eugenol not only acts as an anti‐inflammatory agent but also possesses DNA protective properties (Kumar et al. 2016).

Another study's findings highlighted the critical role of oxidative stress and inflammation in tissue damage, emphasizing the importance of glutathione (GSH) in reversing lipid peroxidation and maintaining cellular integrity. Their findings suggested that clove extract can enhance GSH levels and modulate oxidative stress, which is often linked to neuroinflammatory processes, particularly in neurodegenerative diseases (Ahmad et al. 2012). Most recently, the neuroprotective potential of essential oils, including clove oil, focusing on their ability to modulate microglial activity was demonstrated. The study underscored the dual role of microglia in neuroinflammation and neuroprotection, proposing that targeting specific signaling pathways can mitigate neuroinflammatory responses associated with neurodegenerative diseases. By elucidating the pharmacological mechanisms through which essential oils can influence microglial function, this research provides a compelling argument for the therapeutic application of clove in neuroinflammatory contexts (Stojanović et al. 2024).

#### 3.1.5 Acer Truncatum Oil

Acer truncatum Bunge seed oil (ASO) has garnered attention due to its nutritional and functional significance, particularly due to its high content of unsaturated fatty acids and its role as a primary source of nervonic acid (NA). A previous study conducted an extensive analysis of the oil content and fatty acid composition across various Acer truncatum populations in China, highlighting significant variability in the content of NA, which ranged from 3.90% to 7.85% (Qiao et al. 2018) (Table 1).

Number of studies expanded upon this knowledge by reaffirming ASO's certification as a new food resource by China's Ministry of Health. They emphasized its rich profile of omega −9 fatty acids and the critical role of NA in brain and peripheral nerve tissue maintenance. These articles not only solidified ASO's status as a valuable food oil but also illuminated its potential in functional food development due to its health‐promoting properties (Song et al. 2022; Chen, Song, Song, Cao, et al. 2023).

Most recently, our study presented a comprehensive exploration of the nutritional and economic significance of ASO, particularly emphasizing its fatty acid composition and its implications for neuroprotection. We effectively highlighted the unique profile of ASO, which is characterized by its rich content of omega‐3, omega‐6, and omega‐9 fatty acids, with NA (C24:1, NA) constituting 3%–7% of its composition. This specific fatty acid is particularly noteworthy due to its critical role in the formation and maintenance of brain and peripheral nerve tissues (Song et al. 2022; Chen, Song, Song, Cao, et al. 2023). The study was situated within the context of high‐altitude conditions, specifically focusing on hypoxic‐ischemia encephalopathy (HIE) in neonates. We utilized an oxygen chamber to simulate these conditions, which added a layer of relevance to our findings, given the increasing prevalence of HIE in high‐altitude regions. One of the key strengths of this study is its multi‐omic approach, which allows for a detailed investigation into the molecular mechanisms underlying the neuroprotective effects of ASO. Our findings suggest that administration of ASO can significantly enhance learning and memory capabilities in rats, indicating its potential benefits in cognitive function. Furthermore, ASO not only serves as an important source of essential fatty acids but also possesses anti‐inflammatory and antioxidant properties that are vital for maintaining normal neural transmission and promoting nerve regeneration (Chen, Song, Song, Han, et al. 2023).

#### 3.1.6 Tea Tree Oil

Tea Tree Oil, derived from the leaves of Melaleuca alternifolia. Its primary active component, terpinen‐4‐ol, reveals a comprehensive understanding of its multifaceted therapeutic properties, including anti‐inflammatory and immune‐modulating effects (Ramage et al. 2012) (Table 1).

Number of studies demonstrated the ability of terpinen‐4‐ol to modulate neuroinflammation, which is particularly relevant in the context of neurodegenerative diseases such as AD and PD. These studies highlighted the biochemical pathways through which terpinen‐4‐ol exerts its effects, including the regulation of immune responses, the reduction of oxidative stress and the inhibition of pro‐inflammatory cytokines (Zahedipour et al. 2022; Wojtunik‐Kulesza et al. 2021). This connection between terpinen‐4‐ol and neuroinflammation suggests that tea tree oil could serve as a valuable adjunct in the therapeutic arsenal against neurodegenerative conditions.

#### 3.1.7 Peppermint Oil

Peppermint oil is derived from the leaves of Mentha piperita. Its principal active ingredient, menthol, is responsible for its medicinal properties. Studies surrounding peppermint oil and its primary active compound, menthol, indicate a growing recognition of their potential benefits in neuroinflammatory conditions via exploring various mechanisms through which peppermint oil may exert cognitive and mood‐enhancing effects, primarily through interactions with neurotransmitter systems (Table 1).

It was demonstrated that Peppermint oil could enhance cognitive performance and reduce mental fatigue in healthy adults (Kennedy et al. 2018). These findings highlighted the essential oil's affinity for gamma‐aminobutyric acid A (GABAA) receptors, suggesting that it may facilitate anxiolytic effects, which are particularly relevant in neuroinflammatory contexts where cognitive decline is prevalent (Kennedy et al. 2018). This study set a precedent for further investigations into the cognitive benefits of peppermint oil, particularly in relation to its active components (Kennedy et al. 2018). Building on this, the same researchers reinforced these findings later in 2018, emphasizing the in vitro properties of peppermint essential oils related to neurotransmitter receptor binding and acetylcholinesterase (AChE) inhibition. Their results indicated that higher doses of peppermint oil not only improved cognitive performance but also mitigated fatigue, further supporting its therapeutic potential in cognitive decline scenarios.

A recent study investigated the effects of peppermint essential oil on learning and memory in APP/PS1 transgenic mice, a model for AD. Their findings suggested that menthol enhances learning and memory capabilities. This is a noteworthy angle, as it suggests a potential therapeutic application of peppermint oil in neuroinflammatory conditions characterized by cognitive decline (Lv et al. 2022).

### 3.2 Fatty Acids

#### 3.2.1 Omega‐3 (EPA, DHA)

Previous studies on omega‐3 fatty acids, particularly EPA and DHA, reveal their significant roles in modulating neuroinflammation and enhancing BBB integrity. Research indicated that these fatty acids exert anti‐inflammatory effects by inhibiting the production of pro‐inflammatory cytokines such as IL‐1β and TNFα, which are linked to conditions like neurodegenerative diseases (Logan 2004). The modulation of these cytokines is critical for maintaining neuronal health and mitigating inflammation.

The neuroprotective properties of long‐chain omega‐3 fatty acids were well‐documented, with evidence suggesting that both EPA and DHA play unique yet overlapping roles in the brain (Dyall 2015). These fatty acids are metabolized into anti‐inflammatory mediators, which further contribute to the reduction of neuroinflammation and the preservation of BBB integrity. DHA, in particular, has been identified as a crucial fatty acid for neurogenesis and the resolution of inflammation, with specialized pro‐resolving mediators (SPMs) derived from DHA playing an essential role in promoting anti‐inflammatory responses (Basak et al. 2020).

Moreover, the transport mechanisms that facilitate DHA's entry into the brain are vital for its therapeutic potential. Specific transport proteins, such as fatty acid transport proteins, are crucial for DHA to exert its effects on neural stem cell dynamics and neuroinflammation (Lo Van et al. 2019). The ability of DHA to cross the BBB is essential for its neuroprotective effects, highlighting the importance of effective delivery strategies for these fatty acids in clinical settings.

The competitive inhibition of arachidonic acid (AA) metabolism by EPA and DHA further underscored their anti‐inflammatory properties. By reducing the production of inflammatory eicosanoids derived from AA, omega‐3 fatty acids can significantly lower inflammatory responses (Djuricic and Calder 2021). Additionally, omega‐3 PUFAs have been shown to downregulate NF‐κB, a key transcription factor involved in inflammation, thereby contributing to a more stable BBB and improved neurological health (Zhou et al. 2022) (Table 2).

**TABLE 2:** Summary of fatty acids mechanisms in anti‐neuroinflammation.

Fatty Acids	Component	Possible mechanisms of anti‐neuroinflammation	References
Omega‐3	DHA	Metabolizing into anti‐inflammatory mediators (SPMs); Promoting neurogenesis and inflammation resolution; Enhancing BBB integrity via transport proteins; Inhibiting pro‐inflammatory cytokines	(Chen, Song, Song, Han, et al. 2023; Ramage et al. 2012; Zahedipour et al. 2022; Wojtunik‐Kulesza et al. 2021; Kennedy et al. 2018).
	EPA	Inhibiting pro‐inflammatory cytokines (IL‐1β, TNFα); Competitive inhibition of arachidonic acid metabolism; Downregulating NF‐κB	(Chen, Song, Song, Han, et al. 2023; Ramage et al. 2012; Kennedy et al. 2018)
Omega‐6	GLA	Inhibiting pro‐inflammatory prostaglandins and cytokines; Converting to DGLA for anti‐inflammatory prostaglandin synthesis (e.g., PGE1); Enhancing anti‐inflammatory mediator production; Stimulating inflammation resolution	(Kennedy et al. 2018; Logan 2004; Dyall 2015; Basak et al. 2020; Lo Van et al. 2019)
Omega‐9	OA	Modulating pro‐inflammatory cytokines (IL‐1β, IL‐6, TNF‐α); Promoting anti‐inflammatory cytokine (IL‐10)	(Djuricic and Calder 2021)
	NA	Suppressing proinflammatory cytokine release; Enhancing Aβ clearance	(Zhou et al. 2022)
	EA	Modulating immune responses for anti‐inflammatory; Neuroprotective effects	(Freund‐Levi et al. 2006)

However, the translation of these mechanisms into consistent clinical benefits has proven challenging. Clinical evidence regarding omega‐3 supplementation in neurodegenerative conditions presents a complex picture. While some studies report cognitive benefits in specific patient subgroups, particularly those with early‐stage cognitive impairment (Freund‐Levi et al. 2006; Chiu et al. 2008). Others, including an 18‐month RCT of DHA supplementation, found no significant effect on cognitive decline (Quinn et al. 2010). This discrepancy likely stems from heterogeneity in patient populations, intervention timing, and study design.

#### 3.2.2 Omega‐6 Fatty Acids (GLA)

The exploration of fatty acids, particularly omega‐6 fatty acids like gamma‐linolenic acid (GLA), reveals implications for neuroinflammation and related health conditions. The collective findings from various studies illustrated that GLA plays a significant role in modulating neuroinflammatory responses through multiple mechanisms.

A foundational work established a direct link between omega‐6 fatty acids to the pathogenesis and treatment of MS. Their findings indicated that disturbances in omega‐6 fatty acid metabolism correlated with cytokine dysregulation in MS, highlighting how high doses of GLA‐rich oil could reduce relapse rates and improve disability outcomes (Harbige and Sharief 2007). This establishes GLA as a potent anti‐inflammatory agent, particularly in the context of neurodegenerative diseases. A subsequent study further demonstrated the role of GLA in the context of neuroinflammation by illustrating GLA's ability to enhance the production of anti‐inflammatory mediators, thus reinforcing its role in counteracting neuroinflammatory processes (Dawczynski et al. 2011). The biochemical pathways involved in GLA's anti‐inflammatory effects were elucidated which highlighted GLA's conversion to dihomo‐gamma‐linolenic acid (DGLA) and its subsequent role as a precursor for anti‐inflammatory prostaglandins (Djuricic and Calder 2021). This conversion is crucial as DGLA serves as a precursor for the synthesis of prostaglandin E1 (PGE1), a compound recognized for its anti‐inflammatory properties, particularly in neurodegenerative diseases.

Additionally, a narrative review explored the implications of omega‐6 fatty acids in neurodevelopmental disorders, particularly attention‐deficit hyperactivity disorder (ADHD). They suggested that GLA's anti‐inflammatory properties could play a role in ameliorating symptoms associated with neurodevelopmental disorders. This highlights the broader relevance of GLA beyond inflammatory diseases, extending into mental health and cognitive function (D'Helft et al. 2022). Moreover, another work investigated the mechanisms of neuroinflammation, emphasizing how activated microglia release pro‐inflammatory cytokines, such as IL‐1β, IL‐6, and TNF‐α in the process of neuroinflammation that contribute to neuronal damage. They suggested that fatty acid‐derived mediators, including those from omega‐6 sources could stimulate the resolution of inflammation, thus mitigating cognitive impairment (Tyrtyshnaia et al. 2022). This aligns with the growing recognition of the role of lipid mediators in regulating neuroinflammatory processes (Table 2).

However, the role of omega‐6 fatty acids in neurodegenerative diseases is nuanced and requires a balanced perspective. While some members, like AA, are pro‐inflammatory and may exacerbate disease processes, others such as GLA and linoleic acid (LA) can exhibit neuroprotective potential when used appropriately, particularly in a balanced ratio with omega‐3 PUFAs (Miyake et al. 2010). Clinical studies on the nutritional formula Neuroaspis PLP10, which contains GLA and LA in a 1:1 ratio with omega‐3 s, demonstrated delayed disease progression in Parkinson's disease and reduced relapse rates and disability in multiple sclerosis (Pantzaris et al. 2021). Conversely, observational studies in Alzheimer's disease and amyotrophic lateral sclerosis have not found a protective association for general omega‐6 intake, and a high dietary omega‐6 to omega‐3 ratio is widely considered a risk factor for heightened neuroinflammation (Miyake et al. 2010; Zhu et al. 2021; Fitzgerald et al. 2014). Therefore, the neuroinflammatory impact of omega‐6 fatty acids is not uniform but is fundamentally determined by the specific fatty acid, the clinical context, and, most importantly, the overall balance within the lipidome.

#### 3.2.3 Omega‐9 Fatty Acids (Oleic Acid, Nervonic Acid, Erucic Acid)

The studies on oleic acid, nervonic acid, and erucic acid, prominent omega‐9 fatty acids, reveal their impact on neuroinflammation and immune modulation, indicating their multifaceted role in mitigating neuroinflammatory responses and their potential as a therapeutic agent in various neurological conditions. Research demonstrated that oleic acid could effectively counteract the inflammatory responses typically induced by saturated fatty acids. They reported that oleic acid could enhance insulin sensitivity and modulate the levels of pro‐inflammatory cytokines such as IL‐1β, IL‐6, and TNF‐α while promoting anti‐inflammatory cytokines like IL‐10 (Piccinin et al. 2019). This modulation is essential in creating an anti‐inflammatory environment, particularly within the CNS, where chronic inflammation plays a critical role in neurodegenerative diseases.

Further study presented compelling evidence regarding the role of nervonic acid (NA) in modulating neuroinflammation and its protective effects in the context of neurodegenerative processes. The authors explored the tonic anti‐inflammatory properties of NA, which are particularly relevant in CNS. A critical insight from the study is the ability of NA to suppress the release of proinflammatory cytokines, which are pivotal in the neuroinflammatory response associated with various neurological conditions. This suppression is crucial as excessive cytokine release can exacerbate neuronal damage and cognitive decline. Moreover, the article highlighted that NA promotes endocytosis and the degradation of Aβ, a hallmark of AD pathology (Morgese et al. 2021). This dual action of reducing inflammatory cytokines while enhancing Aβ clearance positions NA as a significant player in mitigating Neuroinflammation and reduction in the risk of neurodegenerative diseases.

A recent study provided a comprehensive examination of the dual nature of erucic acid, particularly in relation to its anti‐inflammatory and neuroprotective properties. The study synthesized a diverse range of studies, including in vitro experiments, in vivo models, and human clinical trials, which collectively illustrate the multifaceted role of erucic acid in neuroinflammation and immune modulation. Notably, the article underscored the potential of erucic acid to mitigate neuroinflammatory responses, which is particularly relevant for various neurological conditions. The authors highlighted that erucic acid's anti‐inflammatory properties may be attributed to its ability to modulate immune responses, thus positioning it as a promising therapeutic agent (Galanty et al. 2023) (Table 2).

The PREDIMED trial demonstrated that supplementation with extra‐virgin olive oil—rich in the omega‐9 fatty acid oleic acid and polyphenols—significantly reduces the risk of major cardiovascular events and stroke. Given the established link between cardiovascular and brain health, coupled with shared underlying pathways involving systemic inflammation, these findings suggest a potential benefit against neuroinflammation, potentially mediated through systemic anti‐inflammatory and antioxidant properties (Stewart 2018). However, clinical research directly investigating the impact of omega‐9 fatty acids on neuroinflammatory biomarkers remains limited, highlighting a critical area for future investigation.

## 4 Clinical Implications and Therapeutic Applications in Neurodegenerative Diseases

Different treatments have been proposed so far for neurodegenerative diseases, but the establishment of their current background has been conditioned by the scarce knowledge about the pathoetiology of these diseases. Many of these treatments have been proved not to have significant effects on the prognosis of patients. Thus, the availability of novel compounds is outstanding in order to find some other alternatives in neurodegenerative treatments. Essential oils and fatty acids appear as promising alternatives to be explored in the field of neurodegenerative processes. There are several current studies focusing on the effect of essential oils and fatty acids on neuroinflammation and neurodegenerative diseases (Barbalace et al. 2023; Sanjay et al. 2022). The methodologies, principal findings, pharmacological doses, route of administration, and the most important outcomes are summarized for each. However, the clinical evaluation of fatty acids and essential oils is hampered by poor experimental design, a lack of efficacy trials, a deficiency in sample size, no long‐term effect studies, as well as low statistical techniques.

### 4.1 Alzheimer's Disease (AD)

Alzheimer's disease (AD) is the most common age‐related neurodegenerative disease. It is recognized by progressive cognitive dysfunction. A characteristic of AD is the development of extracellular Aβ plaques formed by the cleavage of the amyloid precursor protein (APP) by the β‐secretase and the γ‐secretase enzymes. Recent studies have shown that essential oils or fatty acids are able to decrease the production of Aβ (Schwab et al. 2022). It was reported that PUFAs were able to suppress beta‐site amyloid precursor protein cleaving enzyme (BACE) expression in the mouse hippocampus, explaining the beneficial effects of these molecules in AD. Additionally, it was shown that EPA improved cognitive performance and reduces Aβ deposition in a transgenic model of AD, and that DHA provided an increase in memory and Aβ reduction in the brains of animals fed with 1% DHA (Jicha and Markesbery 2010). Therefore, Downregulation of BACE through receptor/PPAR signaling by natural products derived from fish oil, such as EPA and DHA may offer a novel therapeutic strategy to halt or slow the progression of AD. In addition, a comprehensive exploration of the role of essential oils, particularly lavender, rosemary, and ginger in addressing the pathological mechanisms of AD was explored. The authors effectively outlined the critical relationship between neuroinflammation, oxidative stress, and the progression of AD, specifically highlighting how these factors contribute to the accumulation of Aβ and neurotransmission dysfunction (Bavarsad et al. 2023; Sharma et al. 2021).

In a more recent study, a comprehensive overview of the role of omega‐3 long‐chain polyunsaturated fatty acids (LCPUFAs) in the context of AD and aging was reported. The authors highlighted significant findings regarding the dietary intake and plasma levels of omega‐3 fatty acids in individuals with AD compared to cognitively healthy controls. Notably, they reported that Alzheimer's patients exhibit lower levels of DHA in both the brain and cerebrospinal fluid, suggesting a potential deficiency that could be linked to cognitive decline. The article also reviewed observational studies that suggest an inverse relationship between omega‐3 intake and dementia risk, indicating that higher consumption of DHA and EPA may be associated with improved cognitive outcomes. However, the authors critically assessed the results of intervention trials, which have yielded inconsistent findings regarding the efficacy of omega‐3 supplementation on cognitive function in patients with AD. For instance, a meta‐analysis of randomized, placebo‐controlled trials indicated no significant effect of omega‐3 LCPUFAs on dementia severity, quality of life, or mental health in individuals with mild to moderate AD. Specific studies cited in the article demonstrated that supplementation regimens, such as 600 mg EPA and 625 mg DHA daily for 4 months failed to produce meaningful improvements in cognition or mood among Alzheimer's patients. The variability in study designs, dosages, and the specific EPA/DHA ratios administered across trials is notable, reflecting a broader trend in the field where researchers are still attempting to establish optimal conditions for omega‐3 supplementation. The article underscored the necessity for further research to clarify the role of omega‐3 LCPUFAs in cognitive health and the need for standardized protocols in future clinical trials (Troesch et al. 2020) (Table 3).

**TABLE 3:** Therapeutic mechanisms of pharmacological research in neurodegenerative diseases.

Disease	Treatment/therapy	Pharmacological research	References
AD	PUFAs (EPA/DHA) therapy	BACE downregulation via PPAR signaling Aβ deposition reduction	(Harbige and Sharief 2007; Dawczynski et al. 2011; D'Helft et al. 2022; Tyrtyshnaia et al. 2022; Miyake et al. 2010)
	Essential oils (lavender, rosemary, ginger) intervention	Neuroinflammation & oxidative stress modulation	
PD	Dietary DHA Supplementation	Neuroprotection, Anti‐inflammatory, Supports Dopaminergic Function	(Logan 2004; Fitzgerald et al. 2014; Piccinin et al. 2019; Morgese et al. 2021)
	L‐dopa + Natural Antioxidants	Menthol‐Mediated Neuroinflammation Modulation, Neuronal Protection	
	Aromatherapy	Anxiolytic (Lavender), Cognitive Enhancement (Peppermint), Neuroprotective (Rosemary)	
MS	Omega‐3 fatty acid (EPA) supplementation	Immunomodulation (reducing pro‐inflammatory cytokines); Blood‐spinal cord barrier integrity restoration	(Logan 2004; Barbalace et al. 2023; Sanjay et al. 2022; Schwab et al. 2022; Jicha and Markesbery 2010; Bavarsad et al. 2023; Sharma et al. 2021)
	Essential oil therapy (e.g., lavender, rosemary)	Neuroprotection & anti‐neuroinflammation	

At the level of clinical research in AD, different classes of fatty acids reveal distinct roles, with their effects largely mediated through the modulation of neuroinflammatory pathways.

Saturated Fatty Acids (SFA) and Omega‐6 PUFAs are primarily associated with pro‐inflammatory effects that exacerbate AD pathology. Observational studies consistently link higher SFA intake to increased AD risk and accelerated cognitive decline, potentially through mechanisms involving Aβ aggregation and systemic inflammation (Eskelinen et al. 2008; Laitinen et al. 2006). The detrimental impact of specific fats extends beyond individual risk to population health. A large‐scale ecological study across 183 countries established a significant positive linear association between national‐level omega‐6 PUFA intake and the age‐standardized incidence rate of AD (Ciesielski et al. 2025). This macro‐level evidence highlights the potential of population‐wide dietary fat optimization—particularly reducing omega‐6 intake—to mitigate AD burden through anti‐inflammatory mechanisms.

Current evidence from randomized controlled trials indicates that omega‐3 supplementation does not significantly improve cognitive function in patients with established Alzheimer's disease. A meta‐analysis demonstrated no meaningful effect on ADAS‐Cog scores (Kalamara et al. 2025). This conclusion is supported by several large trials: Freund‐Levi et al. found no overall benefit in mild‐to‐moderate AD patients, though a subgroup with very mild disease (MMSE > 27) showed potential improvement (Freund‐Levi et al. 2006). Similarly, Quinn et al. (2010) observed no cognitive protection from DHA supplementation over 18 months (Quinn et al. 2010). A nuanced finding emerged from Chiu et al., where mild AD patients, but not those with moderate disease, exhibited some improvement, suggesting disease severity modifies treatment response (Chiu et al. 2008). Considerable heterogeneity in dosing (240 mg/day to 2.3 g/day), DHA/EPA ratios, and trial duration (3–18 months) complicates direct comparisons. Additional limitations include small sample sizes in some studies and incomplete methodological reporting.

Medium‐chain triglycerides (MCTs) constitute a distinct category with emerging evidence. Unlike long‐chain SFAs, MCTs metabolize into ketone bodies that provide an alternative energy substrate for the brain (Fortier et al. 2021). Short‐term clinical trials indicate MCT supplementation may improve memory function in select AD patients, potentially alleviating the cerebral hypometabolic state linked to neuroinflammation (Fortier et al. 2021). This area nevertheless requires larger, long‐term investigations to establish therapeutic validity.

Collectively, these discrepant findings between supportive observational studies and largely negative randomized trials underscore a critical gap in our understanding. Future research must therefore prioritize well‐designed prevention trials in at‐risk cohorts and resolve the methodological heterogeneity that currently plagues the field.

### 4.2 Parkinson's Disease (PD)

Parkinson's disease (PD) is the second most common neurodegenerative disorder worldwide after AD. It is characterized by the progressive and selective loss of dopaminergic neurons in the substantia nigra pars compacta. Even though the cause of the disease is not known, existing data point to a multifactorial etiology, mainly characterized by the interaction of genetic predisposing factors with environmental agents, such as pesticides, heavy metals, or toxins (De Miranda et al. 2022; Sandeep et al. 2023). Neuroinflammation and oxidative stress have been implicated in the neurodegenerative process of PD, and different studies support the beneficial effects of several essential oils and fatty acids in in vitro and in vivo models of the disease. Nevertheless, only few in vivo animal models or clinical studies have been reported.

A pivotal study highlighted the role of dietary components in mitigating neurodegenerative processes associated with PD. The authors emphasized that dopaminergic neurons are particularly vulnerable to oxidative stress, a condition that is exacerbated in the context of PD. A significant contribution of this study is its exploration of DHA, which has been demonstrated to support dopaminergic function and reduce neuroinflammation. This aligns with the understanding that neuroinflammation is a critical factor in the progression of PD, making DHA a potential therapeutic agent in managing this condition. The authors provided a detailed analysis of how DHA influences neuronal health, suggesting that its incorporation into dietary strategies could be beneficial for individuals with PD. Furthermore, the article discussed the antioxidant and anti‐inflammatory properties of essential oils, specifically peppermint oil. This oil contains menthol, which has been shown to modulate neuroinflammation and protect neurons in various PD models (Angeloni et al. 2017). Building on these insights, another study expanded the discussion by exploring the oxidative toxicity associated with L‐dopa treatment in PD. The study suggests that combining L‐dopa with natural antioxidants, including essential oils may enhance therapeutic outcomes. This combination approach is particularly relevant considering the protective effects of essential oils, which can stabilize cellular membranes and reduce oxidative stress. The positive effects observed with L‐dopa and essential oils in animal models further illustrated the potential for these natural compounds in clinical applications (Nikolova et al. 2019).

Additionally, the exploration of aromatherapy as a complementary therapy for PD has gained traction, particularly in addressing both motor and non‐motor symptoms associated with this complex neurodegenerative disorder. The studies reveal a growing consensus on the potential benefits of essential oils, such as peppermint, lavender, and rosemary, in alleviating the symptoms of PD. However, aromatherapy‐based clinical trials have generally indicated short‐term benefits to some people. For instance, lavender oil is noted for its anxiolytic properties, which could help alleviate anxiety and improve sleep quality in PD patients. Peppermint oil is highlighted for its potential to enhance cognitive function and reduce fatigue, while rosemary oil is discussed in the context of its neuroprotective properties. This exploration of mechanisms not only supports the efficacy of aromatherapy but also provides a scientific basis for its integration into conventional treatment regimens. Moreover, these studies evaluated the methodological aspects of the clinical trials reviewed. While the authors acknowledge the promising results, they also point out the need for larger‐scale studies to establish the efficacy and safety of aromatherapy in PD treatment. They emphasized the importance of rigorous clinical trial designs that account for variables such as dosage, duration of treatment, and patient demographics. This critical stance enhances the credibility of these studies, as it does not simply promote aromatherapy without acknowledging the existing gaps in research (Harbige and Sharief 2007; Yin et al. 2021) (Table 3).

In summary, while the mechanistic rationale for using essential oils and fatty acids in PD is compelling—grounded in their anti‐inflammatory, antioxidant, and putative neuroprotective properties—the current evidence remains predominantly preclinical. The translation of these promising findings into validated clinical benefits is hampered by a scarcity of robust human trials. Existing clinical reports, often small‐scale or focusing on aromatherapy, typically indicate only short‐term or subjective symptomatic relief rather than disease‐modifying effects. Consequently, it is premature to conclude their efficacy in altering PD progression. Future research must prioritize rigorous, large‐scale randomized controlled trials that are specifically designed to evaluate the impact of these natural compounds on core neurodegenerative processes, alongside long‐term safety and optimal administration protocols.

### 4.3 Multiple Sclerosis (MS)

Multiple sclerosis (MS) is a chronic, autoimmune neurodegenerative disease of the CNS, characterized by inflammation, demyelination, and axonal loss. The complex and poorly understood etiology of MS, which involves a multifaceted interplay between autoimmune responses and neuroinflammation, has resulted in a lack of universally effective therapies. In this context, dietary and natural bioactive compounds, particularly omega‐3 fatty acids and essential oils, have garnered increasing attention as potential complementary strategies for managing the disease (Katz Sand 2018; Fathallah et al. 2023).

A growing body of evidence underscores the immunomodulatory properties of omega‐3 fatty acids, such as Eicosapentaenoic Acid (EPA), and the neuroprotective capabilities of various essential oils. Foundational research highlights that omega‐3 fatty acids can modulate immune responses by mitigating the production of pro‐inflammatory cytokines, which are critical mediators in the pathogenesis of MS (Harbige and Sharief 2007).

This foundational understanding was echoed in another work, which presented a clear correlation between erythrocyte membrane fatty acid composition and the clinical manifestations of MS. The authors argued that a deficiency in omega‐3 fatty acids could lead to an imbalance in pro‐inflammatory and anti‐inflammatory cytokines, exacerbating the autoimmune attacks on the myelin sheath. This assertion is supported by previous literature that suggests omega‐3 fatty acids can modulate immune responses, thereby reducing the production of pro‐inflammatory cytokines, which are critical in the pathophysiology of MS. Besides, the article explored the use of dietary interventions, specifically the co‐supplementation of hemp‐seed and evening‐primrose oils, which are rich in omega‐3 and omega‐6 fatty acids. The authors proposed that such dietary strategies could enhance the fatty acid composition in erythrocyte membranes, further promoting a favorable immune response. This aspect of the research underscores the importance of nutritional approaches in the management of MS through their immunomodulatory effects (Rezapour‐Firouzi et al. 2013).

Additionally, Number of studies provided valuable insights into the role of omega‐3 supplementation in MS and experimental autoimmune encephalomyelitis (EAE), an experimental model that recapitulates several of the pathological and clinical aspects of MS. The research conducted on EAE mice reinforced the potential therapeutic implications of omega‐3 supplementation. The studies demonstrated that dietary interventions could prevent the transmigration of macrophages and T‐cells from the sinuses to the perivascular areas of the CNS (Fan and Zhang 2019; Fleck et al. 2021; Fanara et al. 2021; Guerrero Aznar et al. 2022; Kim et al. 2023).

The scope of potential interventions broadens when considering essential oils, such as lavender and rosemary. Preclinical studies suggest these compounds may offer synergistic benefits by reducing neuroinflammation and promoting brain health, presenting a multifaceted approach to MS management (Harbige and Sharief 2007).

However, a critical appraisal of the evidence reveals significant limitations and inconsistencies. The translation of promising mechanistic findings, especially from EAE models, into consistent clinical benefits in humans remains a challenge. Several randomized controlled trials (RCTs) and systematic reviews have reported conflicting outcomes, with some studies finding no significant effect of omega‐3 supplementation on relapsing rates, fatigue, or key inflammatory cytokines compared to placebo (Zandi‐Esfahan et al. 2017; Wergeland et al. 2012). These discrepancies may be attributed to factors such as insufficient sample sizes, short intervention durations, variations in supplement dosage and composition, and the inherent heterogeneity of the MS patient population.

In conclusion, while a compelling theoretical and preclinical basis exists for using omega‐3 fatty acids and essential oils in MS—supported by some clinical evidence for anti‐inflammatory and immunomodulatory effects—the current body of evidence is not yet conclusive. The promising yet inconsistent data underscore the necessity for larger, longer‐duration, and rigorously designed clinical trials to definitively establish their efficacy, optimal dosing, and place in the comprehensive management of MS (Table 3).

## 5 Challenges and Future Directions

Despite the promising potential of essential oils and fatty acids in managing neuroinflammation and neurodegenerative diseases, significant challenges impede their integration into therapeutic practices. Addressing these issues requires concerted efforts in research methodologies, delivery system enhancements, and clinical trial standardization.

### 5.1 Need for Further Clinical Research

Translation of preclinical findings on essential oils and fatty acids to clinical applications is fraught with difficulties. In vivo studies often utilize extremely high concentrations of essential oils to observe anti‐inflammatory effects, which are far beyond achievable physiological levels in humans. Moreover, at physiological concentrations, these oils exhibit limited ability to cross the BBB. The delivery and administration methods employed in previous research are often not feasible for clinical trials. Animal models of neuroinflammation typically rely on acute restimulation or direct administration of inflammatory agents, such as lipopolysaccharide (LPS) in microglial cell cultures, failing to fully mimic the complex, chronic nature of human neurodegenerative diseases. Most critically, the lack of standardized global guidelines for clinical studies means that volunteer and patient data registration lacks uniformity, hindering meaningful comparison and synthesis of research findings. This calls for increased preclinical and clinical investigations, as well as interdisciplinary research to better understand the underlying mechanisms and optimize therapeutic applications.

### 5.2 Potential for Novel Therapies and Drug Development

Recent studies have provided substantial evidence supporting the use of essential oils as adjuvant or primary therapies for neuroinflammatory diseases. Their wide‐ranging anti‐inflammatory properties and diverse bioactive agents make them promising candidates for treating neuropathological conditions. However, practical application is currently limited by the lack of effective systemic drug delivery methods, due in part to the inherent volatility of essential oils. Although the high fat solubility of many of their components allows for systemic administration, conventional drug delivery systems are ill‐suited for essential oils, suffering from low cargo capacity and significant loss of active compounds during micro‐ or nano‐particle synthesis. To overcome these challenges, innovative approaches have emerged, including the use of essential oils in preparing smart materials, micro‐ or nano‐emulsions, and coating conventional delivery vehicles for controlled release at targeted diseased tissues. These advancements offer great potential for future therapeutic interventions but require further refinement and validation.

### 5.3 Standardization and Quality Control

Standardization is a crucial first step in developing drugs from essential oils and essential fatty acids. The chemical composition of these natural products can vary significantly depending on factors such as origin, extraction method, brand, and pharmaceutical form, leading to inconsistent therapeutic outcomes. This variability poses a major obstacle to industrial‐scale production and marketing, necessitating the establishment of strict quality control measures. International standards have been developed, but there is a need for new methods and innovative markers to comprehensively confirm the efficacy and safety of essential oils. Additionally, dedicated guidelines are required to regulate the relationship between manufacturers and sponsors, especially in the context of clinical trials. Adequate funding is essential to implement standardized methods and validate innovative markers, ensuring the reliability and effectiveness of these natural compounds in healthcare.

### 5.4 Potential for Combined Therapies

Given the growing trend of patients using essential oils or dietary fatty acids to alleviate symptoms, evaluating the potential of combined therapies represents an important future direction. Combining these natural compounds with conventional drugs may enhance therapeutic efficacy, reduce reliance on synthetic medications, and extend symptom remission. For instance, the combination of essential oils with conventional analgesics shows promise in enhancing pain management while minimizing adverse effects (Turnaturi et al. 2024). In the context of neuroinflammation, it is imperative to assess the synergistic interactions between essential oils, fatty acids, and conventional drugs to optimize treatment strategies. Comprehensive clinical evaluations are needed to fully understand their multifaceted mechanisms of action and potential synergistic effects, ultimately improving health outcomes for patients with neuroinflammatory conditions.

### 5.5 Neglect of Adverse Effects and Safety (With Verified Supporting References)

The safety profile of essential oils and fatty acid supplements, a critical aspect for clinical translation, has not been adequately addressed in the current literature. While generally regarded as safe, these natural compounds are not devoid of risks. Essential oils, being highly concentrated, can cause adverse effects such as allergic contact dermatitis and skin irritation (Lee 2018; Ramsey et al. 2020). Certain constituents have the potential to inhibit cytochrome P450 enzymes, raising concerns about pharmacokinetic interactions with conventional drugs (Borsini et al. 2021).

Similarly, long‐term high‐dose supplementation with omega‐3 fatty acids is associated with an increased risk of bleeding (Bays 2007). Another potential safety concern is the susceptibility of omega‐3 fatty acid preparations to undergo oxidation, which contributes to patient intolerance and potential toxicity (Bays 2007). Beyond intrinsic biological effects, significant market and quality risks exist due to inconsistent product composition and a lack of stringent regulation. These issues lead to variability in purity and concentration, as well as potential contamination with environmental toxins or oxidized lipids in both fish oil supplements and essential oils (Fialkow 2016).

Therefore, future research and clinical application must integrate rigorous safety assessments, including systematic evaluation of drug interactions, establishment of safe dosing thresholds, and the development of robust pharmacovigilance systems specifically for these natural products. This proactive approach to risk management is indispensable for building a credible and safe therapeutic framework.

## 6 Conclusions

Previous studies indicated that essential oils and fatty acids hold significant promise in transforming the management of neuroinflammation and its associated neurodegenerative diseases, such as AD, PD, and MS through their anti‐inflammatory, antioxidant, and neuroprotective effects. However, further research is warranted to corroborate and expand upon existing evidence. Essential oils such as lavender, peppermint, rosemary, and ginger have demonstrated preliminary potential to modulate inflammatory pathways, reduce oxidative stress, and enhance cognitive function—supporting their candidacy as natural interventions for neurological conditions—though clinical evidence remains mixed. Several trials have reported neutral or inconsistent outcomes, highlighting the need for larger, well‐designed studies to validate these effects. Omega‐3 fatty acids (including EPA and DHA) are essential for maintaining brain health, supporting neuronal function, and mitigating neuroinflammatory markers linked to cognitive decline. While emerging evidence supporting the neurological benefits of essential oils and omega‐3 fatty acids is compelling, critical challenges persist, particularly regarding bioavailability, optimal dosages, and long‐term safety profiles. Collectively, prior research advocates for further investigations into these aspects, with a specific focus on developing advanced delivery systems to enhance therapeutic efficacy. This research direction holds immense promise for improving the management of neuroinflammation and neurodegenerative diseases. Nevertheless, additional large‐scale clinical trials are indispensable to confirm the therapeutic benefits of these natural compounds, establish standardized protocols, and explore their potential in combination therapies.

## Author Contributions

Conceptualization: JinZelong and Songyige. Writing – original draft preparation: JinZelong and Songyige. Writing – review and editing: ChenXianyang and Lijiujun. Supervision: Ahmed Attia Ahmed Abdelmoaty and Lin Feng. All authors have read and agreed to the published version of the manuscript.

## Funding

The authors have nothing to report.

## Consent

The authors have nothing to report.

## Conflicts of Interest

The authors declare no conflicts of interest.

## Data Availability

The authors have nothing to report.
